# The Mediating Role of Perceived Benefit Between Attitudes and Decisional Conflict in Advance Care Planning

**DOI:** 10.1093/geroni/igaf122.2619

**Published:** 2025-12-31

**Authors:** Kyung Ah Cho, Jung-Ah Lee, JinShil Kim

**Affiliations:** Gachon University, Incheon, Republic of Korea; University of California, Irvine, Irvine, California, United States; Gachon University, Incheon, Republic of Korea

## Abstract

Advance directives are essential for ensuring patient-centered decision-making but are often hindered by decisional conflict and varying perceptions of attitudes and benefits. Family caregivers of people with dementia play a critical role in decisions about end-of-life preferences, with their decisional conflict significantly influenced by attitudes and perceived benefits regarding advance care planning. This study examined whether perceived benefits mediate the relationship between attitudes and decisional conflict among family caregivers of people with dementia. A cross-sectional study involving 92 family caregivers was conducted, and mediation analysis was performed using the PROCESS macro for SPSS (Model 4), controlling for age, educational level, and depressive symptoms as covariates. Attitudes toward advance care planning demonstrated a significant relationship with decisional conflict, partially mediated by perceived benefits. The total effect of attitudes on decisional conflict was significant (beta = -1.198, p-value = 0.001), and the indirect effect through perceived benefits was also significant (effect = -0.371, 95% CI = -0.867, -0.033). Additionally, the direct effect of attitudes on decisional conflict remained significant (beta = -0.827, p-value = 0.034). This partial mediation model accounted for 28.2% of the variance in decisional conflict (F = 6.755, R² = 0.282, p-value < 0.001). These findings highlight the importance of perceived benefits in shaping the relationship between attitudes and decisional conflict. Interventions aimed at enhancing positive attitudes and increasing perceived benefits may be essential for reducing decisional conflict, ultimately improving the caregiving experience and decision-making process.

